# Microbial Air Quality in Healthcare Facilities

**DOI:** 10.3390/ijerph18126226

**Published:** 2021-06-09

**Authors:** Lucia Bonadonna, Rossella Briancesco, Anna Maria Coccia, Pierluigi Meloni, Giuseppina La Rosa, Umberto Moscato

**Affiliations:** 1 Department of Environment and Health, Italian National Institute of Health, 00161 Rome, Italy; rossella.briancesco@iss.it (R.B.); annamaria.coccia@iss.it (A.M.C.); pierluigi.meloni@iss.it (P.M.); giuseppina.larosa@iss.it (G.L.R.); 2 Department of Woman and Child Health and Public Health, Fondazione Policlinico Universitario A. Gemelli IRCCS, 00168 Rome, Italy; umberto.moscato@unicatt.it; 3 Section of Occupational Medicine, Institute of Public Health, Università Cattolica del Sacro Cuore, 00168 Rome, Italy

**Keywords:** healthcare facilities, indoor air, microbial contamination, surface contamination

## Abstract

There is increasing evidence that indoor air quality and contaminated surfaces provide an important potential source for transmission of pathogens in hospitals. Airborne hospital microorganisms are apparently harmless to healthy people. Nevertheless, healthcare settings are characterized by different environmental critical conditions and high infective risk, mainly due to the compromised immunologic conditions of the patients that make them more vulnerable to infections. Thus, spread, survival and persistence of microbial communities are important factors in hospital environments affecting health of inpatients as well as of medical and nursing staff. In this paper, airborne and aerosolized microorganisms and their presence in hospital environments are taken into consideration, and the factors that collectively contribute to defining the infection risk in these facilities are illustrated.

## 1. Preface

In healthcare facilities, there is a quite high probability of contracting an infectious disease, whereas the risks associated with acute toxic effects and allergies may be lower [1]. More specifically, the risk of infection becomes prevalent in individuals with an immunodeficiency or immunosuppression that more easily leads to common infections and opportunistic infections, potentially acquired by inhalation and contact. In inpatient rooms, even during medical treatments, biological material from infected individuals can be spread throughout the environment, thereby contaminating the area and promoting the spread of pathogens [2]. In addition, in recent decades, although the use of antibiotics has proved to be an excellent instrument for preventing infections, the widespread use of these antimicrobial substances has inevitably led to the onset of events of microbial resistance to these substances [3].

Healthcare facilities can be considered dynamic environments influenced by a number of factors that collectively make an active contribution to defining the infection risk for patients, healthcare consumers, trainee doctors, temporary visitors, suppliers, contractor staff, administrative and medical personnel and other professionals. The number of occupants, their health conditions and hygiene practices, and the activities they engage in within the facility assume an important role in affecting the overall environment quality. The hygienic conditions of various sites (e.g., reception areas, inpatient wards, administrative offices, etc.), building materials, equipment, and furnishings also affect the composition of the microbial community in hospital environments. All technological systems, such as plumbing, heating, air handling and air conditioning systems, and other equipment can significantly modify the Indoor Air Quality (IAQ), becoming a potential source of bacteria, fungi, viruses and other organisms. Proper design and ongoing preventive maintenance can minimize hazardous conditions [4]. Microclimatic conditions (temperature, relative humidity, and air velocity) and the occurrence of accidental events (water infiltration and condensation) can also promote microbial and fungal growth, leading to harmful indoor conditions. To these factors can be added, in general, external microbial input and seasonal climatic characteristics that affect the microbiological quality of indoor air [5].

In recent years, hospital-acquired infections have constituted a significant cause of morbidity and mortality amongst immunocompromised patients and have also triggered severe medical conditions. The US Centers for Disease Control and Prevention (CDC) estimate that every year in the United States approximately 2 million patients contract an infection in hospital and that approximately 100,000 of them die [6]. However, the true extent of this phenomenon is unknown, due to the difficulties in acquiring reliable and exhaustive data. As a matter of fact, the diagnosis of hospital-acquired infections is complex and based on multiple criteria. World Health Organization (WHO) data show that of every 100 patients admitted to hospital, between 7 and 10 contract at least one infection associated with hospitalization [7].

The main sources of infection in healthcare facilities are patients and healthcare personnel, although the environment itself undeniably plays an important role. Infected patients diffuse microorganisms in the environment by releasing droplets of sputum, fluids secreted by infected wounds, excrement, urine, blood and other body fluids. On the other hand, in addition to pathogenic microorganisms, people harbor an enormous number of microorganisms composing the human microbiome, the complex community of microorganisms living in a symbiotic relationship in human microhabitats. In function of the state of immunodepression of the host, commonly non-pathogenic microorganisms can be a hazard, assuming the role of opportunistic pathogens. Pathogenic or opportunistic agents, such as *Staphylococcus aureus*, *Streptococcus pyogenes* and others can be transmitted also by asymptomatic carriers; this poses important implications for understanding and controlling infectious diseases, especially in healthcare facilities [8].

Although there is limited direct evidence proving that environmental contaminants are the cause of hospital-acquired infections, there is increasing evidence that the environment can act as a reservoir for a number of pathogenic agents and contribute to their diffusion. Besides, microorganisms are often found both on inanimate surfaces, equipment and in the indoor air of environments occupied by colonized and/or infected patients [9,10].

Water distribution systems and the aerosols generated by water cooling systems can also contain pathogenic microorganisms and opportunistic pathogens of purely environmental origin for which plumbing systems provide an ideal habitat (e.g., *Legionella* sp., nontuberculous mycobacteria, and amoebae) [11]. Microbial contamination can also involve drugs during their distribution to patients and food during its preparation, storage and/or distribution. Unless correctly and promptly handled and disposed of, hazardous potentially infected medical waste can also be a source of environmental contamination with serious health consequences [12].

In the air, microorganisms can travel as larger droplets in a short-range, and as smaller aerosols. In this case, they are transported over longer distances by air flows or constitute part of the bioaerosol [13]. Poorly ventilated and /or crowded indoor settings can represent an increased source of spread of microorganisms that can also be also transmitted by direct contact with infectious matter or via contaminated inanimate objects. Although rare in healthcare facilities, direct contact between patients is not uncommon, and it is promoted by unapparent and/or underestimated vehicles of transmission (mobile telephones, earphones, etc.) [14].

However, it is recognized that the hands of healthcare personnel, visitors, and patients can represent the most common vector of hospital-acquired infections [15]. In these settings, hand hygiene must be considered the primary measure for reducing the risk of infections. Numerous studies have also demonstrated that white coats and uniforms worn by healthcare providers are frequently contaminated [16,17].

The microorganisms that can be spread by contact include those associated with conditions such as impetigo, abscesses, diarrhea and scabies and with antibiotic-resistant organisms (methicillin-resistant *Staphylococcus aureus* and vancomycin-resistant enterococci) [18,19]. Transmission by vectors, on the other hand, is limited to areas in which insects, arthropods and parasites are present. One non-negligible aspect regards the antibiotic-resistant bacteria that may be responsible for serious infections associated with medical care. Antimicrobial resistance is still high or is even on the increase in most European countries, especially in the case of common bacteria such as the methicillin-resistant *Staphylococcus aureus* (MRSA), *Escherichia coli*, *Klebsiella pneumoniae* and *Pseudomonas aeruginosa* [20]. In Europe, cases of infection caused by completely or almost completely antibiotic-resistant bacteria have been reported: Carbapenemase-producing entero-bacteriaceae (e.g., carbapenemase-producing *Klebsiella pneumoniae*, CPKP) and multidrug-resistant *Acinetobacter* [21,22,23]. In 2019, an outbreak of carbapenem-resistant New Delhi metallo-beta-lactamase (NDM)-producing *Enterobacteriaceae* occurred in seven hospitals in the same region in Italy, with a total of 350 reported cases (colonized or infected individuals, including 50 cases of invasive disease) [24]. Given the high number of cases, this is an important epidemic event that reveals a shift in the epidemiology of carbapenem-resistant enterobacteria. This change reduces pharmacological treatment options, as the infections associated with the New Delhi metallo-beta-lactamase enzyme do not respond to treatment with some of the new beta-lactam and beta-lactamase inhibitor combinations [25]. In addition, NDM also presents a high risk of diffusion between healthcare facilities.

## 2. Methodology

This review focuses on airborne and aerosolized microorganisms and their presence in healthcare environments; factors that collectively contribute to defining the associated infection risk in these facilities are briefly described.

This narrative review has been carried out through the analysis of papers taken from three main databases: PubMed, SCOPUS, and DOAJ. These latter collect scientific papers from various fields and also from medicine fields, while PubMed contains citations and abstracts from biomedicine and health fields and related disciplines. The review included original papers published in peer-reviewed journals, in addition to reports and guidelines.

The search included the bacterial name plus “healthcare settings”, “indoor”, “air quality” and one or more of the following terms: “outbreak”, “case report”, “infection”, “nosocomial”, and “HAI” (Healthcare-associated infections). A total of 260 papers were found, and 68 articles were discarded because they were outdated or irrelevant.

In order to facilitate the research, since the selection of some specific terms did not permit an adequate sample of identified articles, several searches were made to access an appropriate number of papers.

## 3. Waterborne and Bioaerosol-Associated Infections

In indoor environments, microorganisms can be aerosolized and spread by plumbing systems [26]. As a matter of fact, it has been demonstrated that water and aqueous solutions used in healthcare facilities are often associated with hospital-acquired infections. Despite water treatment and chlorination, water in hospital distribution systems can transport variable concentrations of various autochthonous microorganisms, such as *Pseudomonas* sp., *Legionella* sp., nontuberculous mycobacteria, *Acinetobacter* sp., *Aeromonas* sp, *Sphingomonas* sp., *Enterobacter* sp., *Aspergillus* sp. and free-living amoebae [27]. Incorporated in a matrix of extracellular organic polymers combined with inorganic particles (biofilm), these microorganisms are present in hospital plumbing systems, hot and cold water tanks and cooling towers, as well as in wash basin pipes, shower heads and taps. Although its characteristics depend on certain parameters, and in particular on the specific microbial populations present, the biofilm promotes protection against hostile factors and, at the same time, constitutes a barrier that prevents the total eradication of the microorganisms it contains, with the consequent survival of microbial agents that, through the exchange of genetic material, can also acquire resistance to biocides and antibiotics.

Certain bacteria producing biofilm, such as *Pseudomonas aeruginosa*, *Legionella, Klebsiella, Pantoea agglomerans*, *Enterobacter cloacae* and *Proteus* can cause infections in hospital environments, as they are more resistant to disinfectants and antibiotics than their planktonic forms [28]. Biofilm can act as a microbial reservoir that constantly releases viable microorganisms into the water flow [29]. The aerosol spread from a shower head can potentially contaminate surfaces, medical devices and instruments, as well as endoscopes, dialysis machines, nebulizers, humidifiers and ventilators [30], by means of movement of air in indoor environments. The routes of transmission of waterborne pathogenic agents include direct and indirect contact, use of water for drinking and washing and inhalation of the bioaerosol of contaminated water.

In hospital environments, *Pseudomonas aeruginosa* and *Legionella pneumophila* are the most significant waterborne pathogens [31,32]. *P. aeruginosa* is often associated with hospital-acquired infections, especially among patients on mechanical ventilation or immunocompromised patients in Intensive Care Units (ICUs) [33]. In these environments, it is assumed that the main reservoir of *P. aeruginosa* is the patient’s endogenous flora and that horizontal transmission between patients is the most common source of infection associated with this microorganism. As has been demonstrated by a number of studies, patient-to-patient spread via the hands of healthcare staff and the propagation of the bacterium by means of surfaces are sources of *P. aeruginosa* infection [34,35].

In recent years, the use of molecular typing methods has made it possible to identify a significant source of exogenous strains of P. aeruginosa, isolated from tap water supply in ICUs. A review of prospective epidemiological studies has shown that between 14.2% and 50% of cases of patient infection/colonization was caused by genotypes found in the ICU water supply [33].

The most common form of transmission of *Legionella* is inhalation of contaminated aerosols produced in conjunction with water sprays, jets or mists. Infection can also occur by aspiration of contaminated water, particularly in susceptible hospital patients. Worldwide, waterborne *Legionella pneumophila* is the most common cause of cases, including outbreaks. Already by the end of the 1970s, it had emerged that *Legionella* could be considered a serious risk for immunosuppressed patients, especially in healthcare facilities [36,37,38]. This type of transmission constitutes a considerable risk for patients with chronic lung disease, those undergoing general anesthesia, and all immunosuppressed subjects. In hospital settings, patient immunodeficiency combined with other risk factors results not only in an increased risk of infection, but is also associated with a higher incidence of morbidity than in other types of facility. In 2017, in Italy, of the 2014 reported cases of Legionnaire’s disease, 6.2% involved healthcare facilities. Specific reservoirs of *Legionella* in these environments can be respiratory devices, point of use (showers, taps), humidifiers and cooling towers [39,40].

Non-tuberculous mycobacteria (NTM), also known as environmental or atypical mycobacteria, are responsible for hospital-acquired infections transmitted via inhalation route or direct contact. The structure of their cell wall, which is particularly rich in long-chain fatty acids, and their ability to form biofilm contribute to their extraordinary resistance to chemical substances and allow them to persist in the environment for long periods [41]. Indeed, NTM are commonly found in water distribution systems and they can be aerosolized via showers and taps [42,43]. In this case, a microbiological survey conducted by the authors confirmed the presence of NTM in a hospital plumbing system. Following a number of cases of atypical mycobacteria infection in hospital units, a monitoring study was conducted to identify the sources of risk, to correlate patient exposure with the concentration of NTM in critical points (showerheads and aerators) and to issue corrective measure guidelines. The concentration of NTM observed was between 2 × 10^2^ and 4 × 10^4^ CFU/L and the mycobacteria species isolated and identified included both species of opportunistic pathogens (*M. intracellulare*, *M. chelonae*, *M. llatzerense*, *M. gordonae*) and harmless environmental species [44]. As the risk resulting from the presence of NTM in water cannot be controlled using conventional water disinfection procedures, the installation of filters at the point of use could be the most appropriate option for minimizing exposure.

Hospital water distribution systems can also constitute potential reservoirs of filamentous fungi (molds) such as *Aspergillus* sp., Zygomycetes, *Fusarium* sp., and others [45]. Ubiquitous in nature, molds grow and survive in all types of indoor and outdoor environments. Subjects can be exposed by skin contact, inhalation or ingestion. Inhalation is thought to be the main mechanism of exposure to fungi or their fragments and components [46]. Most fungal spores have an aerodynamic diameter (Da) of 2–10 µm, dimensions that allow them to deposit in both the upper and lower airways of humans [13]. As a rule, severely immunosuppressed subjects have a higher risk of contracting severe fungal infections.

## 4. Air Treatment Systems and Airborne Diffusion of Microorganisms

The airborne infectious particles of microorganisms can take the form of either individual units of infectious load or clusters, usually inside or on the surface of biological matter, known as a “carrier” (the Flügge droplets generated by saliva or nasal mucus and/or pharyngeal or expectorate), or even clusters with polar charges, or that are adsorbed on the surfaces of suspended inert particulate matter [13].

Airborne microorganisms generally do not pose a risk to healthy individuals, whereas pathogenic microorganisms as well as opportunistic environmental agents can be responsible for infections in immunosuppressed subjects in hospital settings. Indoor air quality in healthcare facilities is, therefore, critical and represents an important risk factor.

One of the certainly most important factors affecting indoor air is the efficiency of air handling systems, which can not only be an effective way to prevent airborne disease transmission, but also a way of reducing the spread of chemical and physical contaminants. Besides, air treatment (Heating, Ventilation and Air-Conditioning, HVAC) systems, human activities carried out in the various areas and the presence of subjects with medical conditions can also influence the quality of the circulating air. This is also a consequence of technical choices concerning these systems, sometimes dictated by design choices that are unfit or inadequate for hospital environments, with regard to both the high costs associated with the cleaning of the utility system and the complex or difficult maintenance that make HVAC systems difficult to effectively manage, with a consequent risk for patients and staff [47].

Therefore, air treatment/ventilation systems can capture biological agents through air intake or recirculation (active in many centralized HVAC systems in order to reduce the system’s energetic impact, but extremely hazardous given the tendency to increase the infectious load in environments where high levels of microorganisms are present). Consequently, they can become either a source of production of microorganisms or molds that find, in the particulate matter inside the system’s pipes, an ideal growth substrate, or a means for distant diffusion of contaminants due to the capacities and air flows generated. Inside the ducts, the humidity generated by condensation, together with the dust that accumulates over time, can promote microbial growth and multiplication. Therefore, in hospital environments, specific air management permitting suitable ventilation conditions is of paramount importance. This is particularly important when associated with appropriate filtration and/or the definition of appropriate pressure differentials between environments by means of air flows and turnover identified during the design phase and not subsequently altered in line with changes in the way the healthcare environments are used, as all too often occurs [47].

It has been ascertained that inadequate ventilation is often responsible for the airborne transmission of respiratory viruses [48]. Although the range of diffusion of the microorganisms in air can be very long, it depends on a number of factors, as respiratory activity results in the release of particles of varying dimensions, whose distribution depends on the conditions in which they are released. Whereas infectious particles, as in the case of SARS-CoV-2, with a diameter of between 0.5 and 50 µm (small droplets), can take from hours (>41 h) to seconds (>28 s) to settle within a range of 1 m before falling to the ground, large droplets, with a diameter of 200 µm, take between 2.6” and 0.1” to settle within 1 m from the source. Consequently, these latter represent a minimal or negligible risk of distant transmission, or spread at long distance with HVAC systems. As the probability of a droplet containing virions is proportionate to its volume (therefore to the third power of the diameter), it follows that in the air viruses are in any case carried primarily via “large droplets” that fall rapidly to the ground. Nevertheless, part of the viral units can be released, as mentioned previously, by medium and small droplets that, due to their size, can remain airborne for a long time, thereby forming aerosols of droplet nuclei that can be easily carried by airstreams in indoor environments.

**Figure 1:**
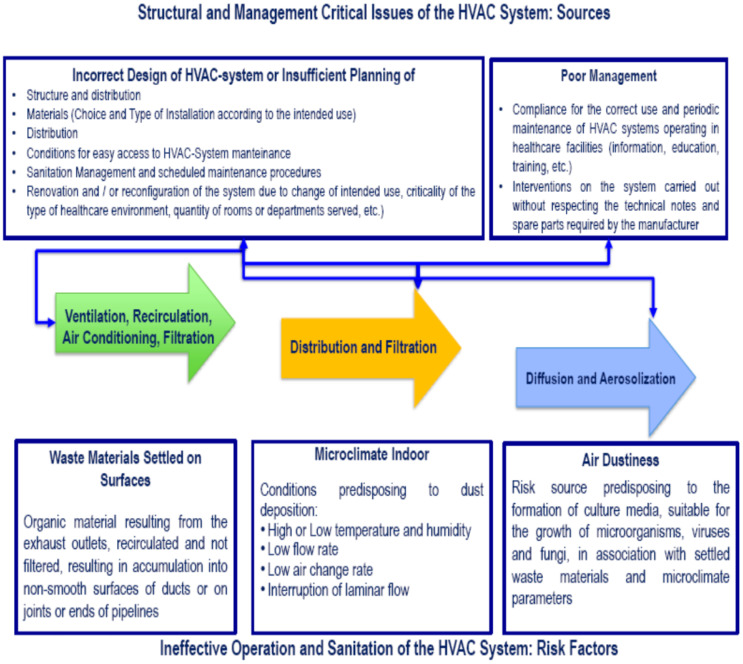
Sources, conditions and risk factors of contamination of the HVAC system: simplified scheme [47].

Droplets larger than 5 μm are primarily produced by coughing, sneezing, singing or speaking. In healthcare facilities, certain medical practices, such as fluid aspiration and bronchoscopy, can also cause the diffusion of particles of this size. The most relevant infections transmitted by droplets are measles, chicken pox, tuberculosis, meningococcal disease, *Mycoplasma pneumoniae*, SARS-CoV-2 (Severe Acute Respiratory Syndrome coronavirus 2), and influenza. Generally speaking, the airborne transmission of infection only regards microorganisms with a low infective dose and takes place following the release of large amounts of microorganisms into the air. The key factors that influence the level of the microbial load in indoor air of a healthcare facility include the number of occupants and the level of relative humidity, which is in turn associated with the specific position of the rooms inside the facility. In general, this influences an HVAC system’s ability to be able to maintain adequate air flow rate and turnover conditions to maintain pressure differentials, when required, especially in critical environments, such as operating theatres and ICUs. This is particularly important considering that the filtration of the systems, despite being applied with high-efficiency filters in healthcare facilities, has not yet shown full efficiency towards those microorganisms—usually viruses—with submicronic dimensions and that are, therefore, smaller than their filtration capacity (e.g., rubella, certain Orthomyxoviruses, etc.). The diagram in Figure 1, provides a simplified overview of critical issues associated with the sources and risk factors for the diffusion of contaminants by HVAC systems [47,49,50,51].

However, it should be noted that healthcare facilities pose an additional problem with regard to the correct management of HVAC systems in order to reduce their impact on the spread of microorganisms in the air. As a matter of fact, the cleaning and maintenance of the units and ducts must be performed with the system switched off and when environments into which the air is released are unoccupied, given the probability of producing dust downstream of the system and consequently contaminating areas with a “dust burst”, despite the implementation of a system decontamination procedure as extensively described in the literature [52,53].

**Figure 2:**
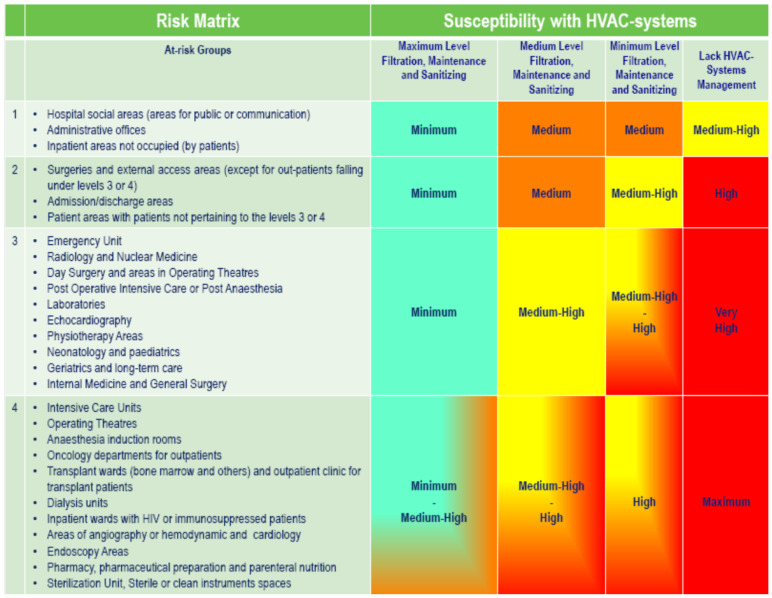
Matrix of the risk level to which the patient is exposed in air conditioned hospital environments [47].

It goes without say that, unlike the case for residential household systems, it is difficult to achieve these conditions in hospital environments, where in emergency departments or ICUs, “shutting down” facilities for several days is not only costly but also impractical. Therefore, in order to express a risk matrix according to hospital environment and the susceptibility of the occupants (remembering that, in addition to patients, healthcare professionals and visitors could present forms of immunodepression), for the management of the cleaning and maintenance of HVAC systems in healthcare facilities, the matrix reported in Figure 2 could be used, considering that cleaning and maintenance status greatly influences the possibility of microorganism spread by or via the units [47,53].

## 5. Mold Infections in Hospital Environments

Molds are often observed indoor in healthcare settings, especially during construction and maintenance works. Fungal spores have a slow sedimentation time and remain airborne for a long time, although they are always present in dust and on surfaces and clothing, even in conditions of low humidity [54,55]. Hospitalized subjects, who have a weak immune response, are more susceptible than healthy individuals to infection by the mesophilic fungi commonly present in nature; in recent decades, high mortality rates have been reported amongst transplant patients and those with leukemia [56,57,58,59,60].

A study conducted following a number of cases of postsurgical infections at a transplant center in Rome included a qualitative and quantitative analysis of the bacteria and molds present indoor and on surfaces of a surgery block (operating theatres, ICUs, surgery ward, treatment rooms and adjacent corridors) [61]. Low mold concentrations were observed in both the air samples and on the surfaces (0–70 CFU/m^3^, and 0–21 CFU/cm^2^, respectively). In addition to the various opportunistic pathogens isolated (*Alternaria infectoria*, *Alternaria tenuissima*, *Epicoccum nigrum*, *Purpureocillum lilacinum*, *Cryptococcus laurentii*), opportunistic molds of environmental origin belonging to the genera *Penicillium*, *Aspergillus*, *Cladosporium*, *Mucor*, *Stemphylium*, *Conidiobolus* and *Trichoderma* were also observed. As regards the bacterial component, the concentrations of biological agents in air varied from 9 to 174 CFU/m^3^, with the highest values observed in the emergency departments. *Staphylococcus aureus* and other opportunistic species of the *Staphylococcus* genus were isolated in many areas. The known opportunistic bacterial species *Leclercia adecarboxylata*, *Enterobacter cloacae*, *Bacillus cereus* and *Kokuria varians* were also detected. In general, moderate microbial contamination was observed on the surfaces examined, with the exception of a high concentration value (>1 × 10^3^ CFU/cm^2^) found on a trolley used for supplying drugs to patients: *Pseudomonas stutzeri*, a known opportunistic pathogen, was detected [61].

## 6. Surfaces as a Potential Source of Infection

In healthcare facilities, beds, sheets, floors, walls, furniture and medical equipment are often subject to microbial colonization able to survive for long periods [62]. Whereas this can represent a limited risk in domestic settings, in healthcare facilities, the presence of immunocompromised patients can constitute an additional health risk. Biological agents, as part of the microbiome of inpatients, may be transmitted to patients by healthcare staff and visitors, via the particulate matter deposited on surfaces and resuspended by the natural process of convection due to air streams and HVAC systems. A room that was previously occupied by a patient colonized or infected by methicillin-resistant *Staphylococcus aureus* (MRSA), vancomycin-resistant enterococci (VRE), *Clostridium difficile*, multidrug-resistant *Acinetobacter* or multidrug-resistant *Pseudomonas*, may constitute an additional risk factor for the newly hospitalized patient [63,64,65].

Among the opportunistic, multiple antibiotic-resistant pathogens detected in hospital settings, the yeast *Candida auris* was first described in 2009. It has been identified as an emerging pathogen and causes candidiasis, currently identified as one of the most common hospital-acquired infections in debilitated or immunocompromised individuals or those undergoing surgery. Its exact route of transmission is still unclear. However, the preliminary evidence suggests that it spreads in healthcare facilities through contact with contaminated surfaces or interpersonal contact. *Candida auris* is an opportunistic pathogen because it can be isolated also in asymptomatic subjects [66]. This yeast has caused a number of epidemics worldwide, and has been reported in Japan, South Korea, India, Pakistan, Venezuela, Brazil, South Africa, Kuwait, USA, Canada, Israel, Britain and Spain, as well as many isolated cases. In the United States, between May 2013 and April 2017, 61 cases of infection were recorded by the CDC, in addition to 32 cases of colonization in asymptomatic subjects. In various outbreaks worldwide, a particularly high mortality rate was observed (30% and 75%). However, many deceased patients already had seriously compromised clinical conditions, which were further complicated by failure to correctly identify the biological agent. In a study conducted in India, 332 samples (32%) collected from different surfaces in a healthcare facility were found to be contaminated: 203 (61%) by Gram-negative bacteria, 216 (65%) by Gram-positive cocci and 52 (16%) by fungi [67]. The most commonly contaminated samples were collected from humidifiers, refrigerators, incubators, medication trolleys, trays and boxes and intensive care equipment. In a pediatric ICU, endemic multidrug-resistant *Klebsiella pneumoniae* was frequently isolated [68,69].

The main hospital-acquired pathogens able to survive on inanimate surfaces, and the duration of their persistence are listed in Table 1 and Table 2. In most cases, humidity improves the persistence of different types of bacteria (e.g., *Chlamydia trachomatis*, *Listeria monocytogenes*, *Salmonella typhimurium*, *Pseudomonas aeruginosa*, *Escherichia coli*), whereas *Staphylococcus aureus* alone survives longer at lower humidity values. In the environment, Gram-positive bacteria survive longer than Gram-negative bacteria [70,71,72,73,74].

The immune status of subjects in the highest infection risk areas and the procedures they undergo make patients particularly vulnerable to microbial infections. Inside a surgery block, microbial contamination can be primarily attributed to the airborne microorganisms convey by surgical staff and patients; other potential sources of contamination are controlled-contamination HVAC systems and non-sterile instruments, which have an impact on environmental conditions. In this context, the transmission of *Mycobacterium chimaera* in hospital environments, a bacterium found in biofilm and tap water, seems to be correlated with the introduction in areas at risk of medical instruments and surgery devices. With regard to specific appliances, there have been recent reports of cases of invasive cardiovascular *Mycobacterium chimaera* infections. *M. chimaera* can cause lung infections, especially in immunocompromised patients [70,71]. The first report on *M. chimaera* infections following heart surgery infections was published in 2013 [72]. Other studies described cases of *Mycobacterium chimaera* infection with endocarditis of the artificial heart valve and infections of the vascular graft and an epidemiological connection was established with the heater-cooler units (HCU) used during the surgical procedure [73]. Since then, further cases of *M. chimaera* have been reported associated with the use of these systems in patients undergoing open heart surgery in various European countries (France, Germany, Ireland, Holland, Spain, United Kingdom and Switzerland, as well as the United States, Canada, Australia and Hong Kong; the first report in Italy was in June 2018) [74]. Heating-cooling units are classified as class IIb medical devices and are used during cardiothoracic surgery procedures involving the heating/cooling of the patient. These devices consist of tanks that supply water at a controlled temperature to heat exchangers and to heating/cooling blankets, via water circuits. To date, it has not been possible to identify the equipment’s exact role in transmitting *M. chimaera* to the environment. However, the manufacturers of the equipment have issued guidelines and safety warnings to operators.

## 7. Conclusions

Healthcare-associated infections (HAIs) have a special place among the risks associated with health and social care, due to their size, complexity of determinants, and epidemiological trends. The clinical-economic impact is significant: according to a WHO report healthcare-associated infections cause prolonged hospital stays, long-term disability, increased antibiotic microbial resistance (AMR), additional costs, and excess mortality [185].

Cassini et al. estimated that 2,609,911 new cases of HAIs occur every year in the European Union and European Economic Area [186]. In Italy, the number of HAIs would appear to vary from 450,000 to 700,000 per year, with 30% of infections being preventable [187].

The role of the hospital environment in the transmission of HAIs is still a matter of debate in scientific communities. In fact, the global burden of healthcare-associated infection is unknown because of the difficulty of gathering reliable diagnostic data. However, evidence seems to confirm that healthcare settings represent a large reservoir of pathogenic and opportunistic microorganisms from different matrices such as air, surfaces, medical equipment and water systems. Moreover, spread of pathogens can result from inpatients themselves, visitors, and healthcare personnel. In addition, the use of antibiotic therapies causes the selection of multi-resistant pathogens that spread within the facility, increasing the risk for exposed individuals.

Despite efforts to implement preventive measures, it is still difficult to record HAIs according to the source of infection.

However, it can be argued that water is one of the most reported source of infection because of possibility to verify its quality. Water safety in healthcare settings is a top priority and a constant challenge for these facilities. Control and management of water quality issues in healthcare facilities is a topic of great interest and certainly intersects with assessment and management of air quality. In fact, aerosolization of contaminated water from showers, faucets, and medical devices poses a real health risk in settings such as hospitals and nursing homes. In addition, it is recognized that the quality standards prescribed to date by water quality legislation have not been able to ensure the safety of particularly vulnerable populations or individuals. In fact, they are requirements aimed at ensuring the quality of water intended for consumption by a healthy population. Nevertheless, it is known that colonization of plumbing or ventilation systems by *Legionella* can often cause Legionnaires’ disease in healthcare facilities. Exposure to the risk of acquiring the disease occurs through the airways as a result of inhalation of aerosols of contaminated water released from showers, air humidifiers and medical devices, including dental equipment [188].

Conversely, among the countless sources of HAIs, one of the most difficult environmental components to study and assess is certainly air from a microbiological perspective, and still more in healthcare facilities where several factors have a large impact on the air quality.

The need for multiple strategies to control the spread of pathogenic microorganisms and adoption of appropriate preventive measures could allow identification of the real role that healthcare settings have in the spread of infections. Nevertheless, at an international level, there is no consensus regarding the methods to be adopted in order to measure and analyze airborne biocontamination, in particular in high-risk areas, and official methods and frequency of sampling and analysis cannot be universally adopted for each circumstance. These difficulties reflect the current situation: although there are recommendations from international agencies and institutions, there are no legislative values or health-based standards for the microbiological parameters of indoor air quality due to the difficulties encountered in associating the data of the microbiological tests with those of epidemiological investigations.

A safe environment plays an important role in the prevention of HAIs and the spread of AMR. Many factors, including the design, organization and management of the healthcare facility, availability for safe water, appropriate sanitation, air quality and efficiency of the air treatment equipment, can significantly influence the transmission of infections. Many infection prevention and control measures, including hand hygiene, are simple, low-cost and effective; however, they require awareness by healthcare providers and personnel.

From the literature, the need for an efficient control of microbial contamination on surfaces in hospital environments even strongly emerges. Indeed, these surfaces can easily represent significant sites of colonization from microorganisms that may contribute to the transmission of HAIs.

Control of surface bacterial load is routinely addressed with the use of conventional chemical-based soap/disinfectants. However, these can be ineffective in preventing recontamination and can select strains resistant to disinfectants themselves. Recently, cleaning agents containing probiotic agents have been proposed for hospital sanitation and have been shown to stably reduce surface pathogens up to 90% more than conventional disinfectants [189].

Infection control programs are defined by the WHO and CDC [185,190,191,192]. Improving HAI monitoring systems and implementing standard procedures to reduce microbial spread in higher-risk areas should be the primary goals, especially during public health emergencies.

## Figures and Tables

**Table 1:** Pathogenic bacteria and fungi detected in fomites and hospital environments, their survival and references.

Bacteria	Duration of Survival on Inanimate Surfaces (Range)	References
*Acinetobacter baumannii* (including MRD)	2 days–>4 months	[75,76,77]
*Bordetella pertussis*	3–5 days	[78]
*Burkolderia cepacia*	>1 week	[79]
*Campylobacter jejuni*	15 min–7 h	[80]
*Chlamydia pneumonae/tracomatis*	30 min–≤30 h	[81,82]
*Chlamydia psittaci*	15 days to months	[83]
*Clostridium difficile* (spores)	5 months	[84]
*Corynebacterium diphtheriae*	7 days–6 months	[78]
*Corynebacterium pseudotuberculosis*	1–8 days	[85]
*Enterococcus faecalis/faecium* (including VRE)	5 day–3 months	[86,87,88,89]
*Escherichia coli* (including pathogenic)	<1 h–28 days	[77,90,91]
*Haemophilus influenzae*	12 days	[9]
*Helicobacter pylori*	≤90 min	[92]
*Klebsiella pneumoniae*	1 h–6 weeks	[93,94,95,96]
*Listeria monocitogenes*	at least 24 h	[97]
*Mycobacterium tuberculosis*	10–120 day	[98]
*Neisseria gonorrhoeae*	>24 h	[99]
*Neisseria meningitides*	72 h	[100]
*Proteus mirabilis*	4 h–26 days	[94]
*Proteus vulgaris*	1–2 days	[98]
*Pseudomonas aeruginosa*	5 h–33 days	[93,101]
*Salmonella enterica* serovar Abony	1–>24 h	[102]
*Salmonella enterica *serovar Enteritidis	1–48 h	[93,102]
*Salmonella enterica* serovar Typhimurium	5 h–12 weeks	[93,103,104]
*Salmonella typhi*	6 h–4 weeks	[98]
*Salmonella* spp.	at least 30 days	[105]
*Serratia marcescens*	<1 h–11 days	[93,94]
*Shigella* spp.	1.5–4 h	[106]
*Staphylococcus aureus* (including MRSA)	6 h–12 days	[77,107,108]
*Stenotrophomonas maltophylia*	2–7 days	[101]
*Streptococcus pneumoniae*	1 day up 30 months	[109,110]
*Streptococcus pyogenes*	3 days–6.5 months	[110,111]
*Yersinia pestis*	up to 5 days	[112]
Yeasts and Molds		
*Candida albicans*	1–120 days	[113,114]
*Candida auris* (MRD)	3–14 days	[114,115,116,117]
*Cryptococcus neoformans*	30 days	[101]
*Aspergillus* spp. (conidia)	1 year	[118,119]
*Aspergillus flavus*	2–30 days	[120,121]
*Aspergillus fumigatus*	1–30 days	[101,120,121]
*Aspergillus niger*	1–30 days	[120,121,122]
*Fusarium* spp.	48 h–>30 days	[120,121,123]
*Mucor* spp.	16–>30 days	[121]
*Paecilomyces* spp.	<1–11 days	[120]
*Penicillum crysogenum*	6–120 h	[121]

MRD: multi resistant drugs.

**Table 2:** Viruses detected in fomites and hospital environments, their survival and references.

Viruses	Duration of Survival on Inanimate Surfaces (Range)	References
Adenovirus	1 h–>12 weeks	[124,125,126,127,128,129,130,131]
Astrovirus	7–90 days	[126]
*Caliciviridae*	<5 min–>168 days	[132,133,134,135,136,137]
Coronavirus (SARS-CoV-2, SARS-CoV, MERS-CoV, HCoV -229E, HCoV -OC43, HCoV -NL63)	30 min–>8 days	[131,138,139,140,141,142,143,144,145,146,147,148,149,150]
Coxsackie virus	2–5 weeks	[125,130,151]
Cytomegalovirus	1 h–4 h	[152,153]
Echovirus	48 h–7 days	[127,154]
Filoviruses	2 days–>32 days	[155,156]
Hepatitis A Virus	2 h–>60 days	[126,129,132,157]
Hepatitis B Virus	>14 days	[158,159]
Hepatitis C Virus	5 days–6 weeks	[160,161,162]
Human Immunodeficiency Virus	>5 days	[163,164,165]
Herpes Simplex Virus, Type 1 and 2	4.5 h–>8 weeks	[125,130,131,151,163] [166]
Human Metapneumovirus (HMPV)	2–8 h	[167]
Influenza Virus	1–2 days	[127,147,168,169,170,171,172]
Parainfluenza Virus	<0.5 h–>8 h	[169]
Feline Norovirus and Calicivirus	8 h–7 days	[133,134]
*Papillomaviridae*	>7 days	[173]
Papovavirus	8 days	[163]
Parvovirus	>1 year	[163]
Poliovirus Type 1	4 h– >60 days	[129,154,157,163,174]
Poliovirus Type 2	1 day–8 weeks	[125,126,130]
*Poxviridae*	<1 day–56 days	[125,175]
Pseudorabies Virus	>/= 7 days	[176]
Respiratory Syncytial Virus	0.5 h–6 months	[177]
Rhinovirus	2 h–7 days	[178,179]
Rotavirus	1 h–>60 days	[126,129,180,181,182,183]
Vaccinia Virus	3–>20 weeks	[125,184]

## Data Availability

No datasets were generated or analyzed during the current study.
